# Speech Enabled Reading Fluency Assessment: a Validation Study

**DOI:** 10.1007/s40593-025-00480-y

**Published:** 2025-05-14

**Authors:** Max van der Velde, Wieke Harmsen, Bernard P. Veldkamp, Remco Feskens, Jos Keuning, Nicole Swart

**Affiliations:** 1 Cognition, Data and Education Section, Faculty of BMS, University of Twente, De Zul 10, Enschede, NJ 7522 The Netherlands; 2 CitoLab, Cito, Arnhem, The Netherlands; 3 Expertisecentrum Nederlands, Nijmegen, The Netherlands; 4 Centre for Language Studies, Radboud University, Nijmegen, The Netherlands

**Keywords:** Validation, Reading, Fluency, Assessment, Instrument, Automatic

## Abstract

Although the ability to comprehend what one is reading is one of the most fundamental necessities to function within society, the reading comprehension skills of students have recently been on the decline in many countries. An essential prerequisite to reading comprehension is the ability to read fluently, which is defined as the ability to read (aloud) with accuracy, speed, automaticity and prosody. Current oral reading fluency assessment instruments seldom provide detailed diagnostics however, and bestow a heavy testing burden on practitioners. Recent developments in Artificial Intelligence-based assessment methodology might provide a solution to current assessment issues, but thorough validations of such procedures have proven scarce. This study evaluates whether valid word decoding and passage reading measures (accuracy, speed and automaticity) can be generated for a semi-transparent language, using an automatic speech recognition (ASR) based oral reading fluency assessment instrument. A validation study was conducted, using the Argument-Based Approach to Validation. Data concerned 176 h of speech data, and the results of 569 and 622 oral word- and passage reading tests that are currently administered in primary schools, from 653 children attending the second- or third grade of Dutch primary education. The results of the validation indicate that it is possible to generate fluency metrics for a semi-transparent language, using an ASR-based oral reading fluency assessment instrument. Future researchers are advised to further optimize the ASR, evaluate its errors, and realize a prosody component, completing the envisioned reading fluency assessment instrument, thereby improving reading fluency assessment throughout primary education.

**Supplementary Information:**

The online version contains supplementary material available at 10.1007/s40593-025-00480-y.

## Speech Enabled Reading Fluency Assessment: a Validation Study

Being able to comprehend what you are reading is one of the most fundamental necessities to function within society. Nevertheless, the reading comprehension skills of students have recently been on the decline in many countries (Meelissen et al., 2023; Mullis et al., 2023), increasing the risk of functional illiteracy among future generations. One of the most important prerequisites to reading comprehension is the ability to read fluently (Fuchs et al., 2001; Hoover & Gough, 1990), which is often defined as the ability to read aloud with accuracy, speed and proper expression (Kuhn et al., 2010; Pikulski & Chard, 2005). Indeed, the importance of fluency to comprehension is so well-established that some interventions aimed at improving comprehension have focused on improving fluency instead (Mastropieri & Scruggs, 1997; Reutzel & Hollingsworth, 1993). While it is not suggested that reading fluency interventions should substitute for specialized reading comprehension training, such implementations do demonstrate the relevance of fluency to comprehension.

To elaborate, research has increasingly linked comprehension to the automaticity and prosody of reading (Groen et al., 2018; Kuhn et al., 2010). Here, automaticity reflects the ability to decode written text with sufficient accuracy and speed (e.g. Kim et al., 2021), which a reader attains through instruction and practice (Logan, 1988). Automatic reading reduces the mental effort required to read, allowing the reader to focus on more cognitively demanding tasks, like comprehension (Aldhanhani & Abu-Ayyash, 2020; Morris & Perney, 2018). To elaborate, Perfetti’s (1985) verbal efficiency theory states that lower complexity tasks must be mastered to some degree, before more complex tasks can occur during reading. Correspondingly, automaticity can be related to comprehension through proficiency in decoding, word identification and the retention of limited cognitive resources (Perfetti & Stafura, 2014).

Meanwhile, prosody reflects expressive components of reading, such as phrasing, expression, intonation, stress and pitch (Miller & Schwanenflugel, 2008; Share, 2008), which facilitate or enhance the retention of meaning (Kuhn et al., 2010; Miller & Schwanenflugel, 2008; Silva et al., 2021). Prosody and comprehension have been shown to affect one another throughout most of primary education (Veenendaal et al., 2016), even when controlling for automaticity (Groen et al., 2018; Veenendaal et al., 2015). In short, the relationship between fluency and comprehension has been well-documented and involves automaticity and prosody.

In contrast, standardized oral reading fluency assessment tools, such as Dynamic Indicators of Basic Early Literacy Skills Oral Reading Fluency (DORF; University of Oregon, 2020), and the Test of Word Reading Efficiency Sight Word Efficiency (TOWRE-SWE; Torgesen et al., 1997), assess fluency using the number of words read correctly per minute (WCPM). This metric only integrates measures of accuracy (words read correctly) and speed (per minute), thereby solely reflecting automaticity. Meanwhile, prosody is separately measured through subjective rating scales (Kuhn et al., 2010; Morrison & Wilcox, 2020), that require multiple trained raters to obtain reliable results.

In practice, the time-consuming nature of oral reading fluency assessment, the requirement for training, and the resulting testing burden, has led teachers and other practitioners to relinquish the assessment of prosody. In addition, practitioners stray from obtaining detailed automaticity diagnostics, as their extraction further increases assessment duration. This lack of complete and detailed diagnostics is problematic, as it complicates the implementation of individualized reading instruction, which has increasingly been identified as crucial for the optimal development of individual readers (Bray & McClaskey, 2015; Connor & Morrison, 2016).

To summarize, although fluency is a crucial prerequisite for developing reading comprehension skills, its assessment seldom supplies practitioners with detailed diagnostics, and bestows a heavy testing burden upon them. Therefore, an assessment instrument that provides detailed individualized diagnostics, and that reduces the testing burden placed on practitioners, could improve the development of reading fluency and comprehension alike. To fulfill this ambition, a recent review on oral reading fluency assessment has suggested the use of artificial intelligence-based speech technology (van der Velde et al., 2024a), an approach that has served a multitude of scientific fields.

Over the last two decades, major advances have been made regarding the availability and complexity of artificial intelligence-based technology, increasing their applicability and relevance for assessment (Clarke-Midura & Dede, 2010). For example, developments in text processing have allowed for the automatic evaluation of theoretical papers (Rokade, 2018). Likewise, the development of automatic speech recognition (ASR), which concerns the “independent, machine-based process of decoding and transcribing oral speech” (Levis & Suvorov, 2012, p. 1), has made audio data a valuable source of information.

With regard to reading fluency, the effectiveness of ASR has been demonstrated in early work by Mostow et al. (2003) and Reeder et al. (2007), while the current relevance of ASR is reflected through its central role within recent reading fluency assessment frameworks (Silva et al., 2021). Correspondingly, much work has been conducted to automate fluency assessment through ASR, especially for English readers (Cheng & Shen, 2010; Loukina et al., 2019; Sabu & Rao, 2018). This, in turn, has led to the creation of automatic reading fluency tools such as the Fluent Oral Reading Assessment (FLORA; Bolaños et al., 2013), and Moby.Read (Cheng, 2018).

Even though the body of literature that implements ASR within the English language is substantial, this does not necessarily indicate that these results are generalizable to all languages. Namely, although most literary research focusses on English, English has long been established as an outlier orthography (Share, 2008). Specifically, English has relatively high irregularity, or low transparency, with regard to grapheme-phoneme correspondences when compared to other languages. As previous research has illustrated, the transparency of a language impacts the way in which children process, learn, and attempt to express a language (Smith et al., 2021; Wimmer & Goswami, 1994). For example, children learning less transparent orthographies favor the direct recognition of words or letter-strings over converting graphemes into phonemes.

Throughout the last decade, attempts have been made to implement ASR within non-English languages. For example, Proença et al., (2015, 2017) automatically evaluated disfluencies in the speech of Portuguese children. Another example concerns the Dutch language, where crucial steps have been made regarding the automation of assessment of first graders (Bai et al., 2020, 2021), and secondary language learners (Cucchiarini et al., 2009; Wei et al., 2022). However, less attention has been placed on children attending Grades 2 and 3, even though these prominently featured in the reading fluency assessment literature (van der Velde et al., 2024a). In addition, current ASR implementations primarily focus on accuracy, ignoring speed and prosody.

To overcome current assessment shortcomings, and to investigate the full potential of ASR based fluency assessment, an assessment instrument that utilizes automatic speech recognition to provide detailed diagnostic information on all fluency components has been developed (van der Velde et al., 2024b).

### SERDA: Automatic Oral Reading Fluency Assessment for Dutch

The Speech Enabled Reading Diagnostics App (SERDA) is a Dutch oral reading fluency assessment instrument, developed to improve reading education at the primary school level (van der Velde et al., 2024b). Through the incorporation of speech recognition, speech-based diagnostics, and their conversion into didactic suggestions for practitioners, SERDA allows for the provision of individualized feedback on children’s oral word and passage reading performance, as well as detailed information on all fluency components. All the while, SERDA’s short administration duration and automatic scoring should reduce the testing burden placed on teachers and other practitioners.

Although these findings are promising, the ASR-based accuracy, speed, automaticity and prosody metrics of any automatic fluency instrument should be thoroughly validated before statements can be made about their usability in practice. Given that reading fluency is currently assessed through the speed, accuracy and automaticity of reading in practice, we argue that it should first be proven that ASR-based metrics can validly substitute for their pen-and-paper contemporaries, before prosody is considered. Therefore, the current study will focus on validating SERDA’s word decoding and passage reading tasks, excluding prosodic metrics, to determine whether an ASR-based reading fluency assessment instrument can provide valid word decoding and passage reading metrics.

### Validating ASR-Based Decoding Scores

To substitute for current instruments, an ASR-based decoding instrument should provide observable and reliable scores based on children’s oral reading performance. Moreover, the ASR-based scores, obtained over a limited sample of reading items and primary school children, should be generalizable to all potential samples of reading items and primary school children. Furthermore, the scores should be proven to reflect oral reading skills, allowing for claims to be made regarding children’s oral reading performance. Finally, the reading tasks should provide scores that allow practitioners to differentiate between good, average and less proficient oral readers, such that decisions with regard to development and proficiency can be made.

In order to evaluate whether these requirements are met, we will apply the Argument-Based Approach to validation (ABP; Kane, 1992, 2006, 2013). Within the ABP, an Interpretation and Use Argument (IUA) is specified, which describes the inferences and assumptions underlying the proposed interpretation of assessment results. Then, a validity argument is defined, describing the process of evaluating the components of the IUA through gathered evidence. Lastly, the validation as a whole is evaluated. Specifically, it is evaluated whether the correct assumptions and inferences are addressed, whether the inferences can be justified, and whether the validity argument, as a whole, is plausible.

### The Present Study

The present study aims to evaluate whether it is possible to generate valid word decoding and passage reading measures for a semi-transparent language, using an ASR-based oral reading fluency assessment instrument.

Based on the ABP framework, the main question answered with this study is:

- Can an oral reading fluency assessment instrument that utilizes automatic speech recognition provide valid word decoding and passage reading scores?

In order to answer this question, we will answer the following sub-questions:

1. Can performances on the reading tasks be translated into observable and reliable ASR-based scores?
2. Is the sample of reading tasks and primary schools representative of the population of reading tasks and primary schools?
3. Do the ASR-based scores reflect oral reading skills, allowing for claims to be made regarding children’s oral reading performance?
4. Can the ASR-based oral reading scores differentiate between good, average and less proficient oral readers, such that they can be used to make decisions regarding their proficiency and development?

## Methods

To determine whether an ASR-based oral reading fluency assessment instrument could provide valid word decoding and passage reading scores, we administered SERDA’s word- and passage reading tasks (van der Velde et al., 2024b), as well as the most popular instruments to monitor oral word and passage reading skills in the Netherlands: the Three Minute Task [*Drie-minuten-toets*; DMT] (van Til et al., 2018a) and AVI [*Analyse van Individualiseringsvormen*; AVI] (van Til et al., 2018b).

### Participants

One hundred seventy-six h of speech data were obtained, as well as the results of 569 DMT and 622 AVI administrations, from 653 (52% girls) children attending the second and third grade of Dutch primary education. Children attended 19 different primary schools, selected to represent dialect regions (Cucchiarini et al., 2008) and school-weights, an indicator of expected school performance and social economic status of children’s parents (Inspectorate of Education, 2024). The average age of the children was seven and a half (*SD* = *0.74)*, with children attending Grade 2 being one year younger, on average, than children attending Grade 3.

### Materials

#### SERDA: Word and Passage Decoding

SERDA’s word and passage reading tasks were individually administered on a tablet, during which children’s speech was recorded through a microphone. The word decoding task contained 150 words, chosen based on the DMT (van Til et al., 2018a) and expert opinion. Words were divided over three 50-word subtasks, which varied with regard to the number of syllables per word and the complexity of reading difficulties. The presentation of words followed a progressive demasking design (Grainger & Segui, 1990) to allow for accurate reading speed estimation. During the progressive demasking task, a mask was placed over the words at an increasing interval, such that the to be read word became visible for longer over time, until the participant indicated that they were able to recognize the word. Before administration, children were instructed to tap the screen as quickly as possible once the presented word was recognized, after which they read the word out loud as accurately as possible.

The passage reading task contained three passages of about 175 words, which were constructed using the guidelines of the AVI (van Til et al., 2018b). The passages were written by children’s authors, discussed topics of interest to children, and contained multi-syllable words with reading complexities that corresponded to those, respectively, expected at the end of second grade, the middle of third grade and the end of the third grade. Children were instructed to read the passages as quickly and accurately as possible, including the title. The task was finalized once the entire passage was read, or after 3 min had passed.

Each word and passage reading subtask yielded audio- and log files, based on which item-, subtask- and person-level measures were extracted. An overview of the extracted measures can be found in Table 1. To extract these measures, the same methodology was adopted as described in van der Velde et al. (2024b), using an updated version of the ASR model. In addition, we utilized Item Response Theory (Hambleton & Swaminathan, 1985) to extract item- and person parameters. Specifically, we applied the Hierarchical Bayesian joint modeling approach (van der Linden, 2007), which allows for the joint modelling of accuracy and speed scores, integrating both accuracy and speed information during the estimation of children’s oral reading skills. Here, we used the “LNIRT” (Fox et al., 2021) R-package to obtain item difficulty and discrimination parameters for all items. Finally, we calculated LNIRT word decoding and passage reading ability and speed estimates for each person, using their item-level accuracy and speed scores.

**Table 1:** Item, Subtask and Person Level Measures Extracted by SERDA

Measure	Word Decoding	Passage Reading
Item level		
Accuracy	0 or 1	0 or 1
Speed	Flashing time (seconds)	Speaking duration (seconds)
WCPM	-	-
Subtask level		
Accuracy	Number of words read correctly	Number of words read correctly
Speed	Words read divided by total flashing time	Words read divided by task duration
WCPM	Accuracy divided by total flashing time	Accuracy divided by task duration
Person level		
Accuracy	Average subtask-level accuracy	Average subtask-level accuracy
Speed	Average subtask-level speed	Average subtask-level speed
WCPM	Average subtask-level WCPM	Average subtask-level WCPM

Adapted from van der Velde et al. (2024b)

Before model specification, words or items with little to no variability and persons with extremely unlikely scores or mostly missing data were removed. For the word decoding task, we retained observations of 633 children for 149 items. For the passage reading task, we retained observations of 631 children for 526 items.

#### Word Decoding: Three Minute Task [Drie-minuten-toets; DMT]

The DMT is an on-paper examination, aimed at monitoring the development of word decoding skills of children during Grades 1 to 6 of primary education (van Til et al., 2018a). DMT administrations were individually conducted and scored by teachers of the schools the children attended. Children read up to three word lists of increasing difficulty, as quickly and accurately as possible, for a duration of one minute per list. Then, based on their performance compared to grade-specific norms for the population of primary schoolers, children were classified into one of five categories, ranging from the 20% best to least developed readers. As these categories were provided by school after specification, no reliability or validity information was collected. However, the reliability and validity of the DMT has previously been thoroughly investigated (Van Til., 2018a).

Based on the DMT classifications, we specified three proficiency classes. To elaborate, children in the 20% least developed DMT group were classified as “Less Proficient”, while children classified into the top 20% were classified as “Highly Proficient”. Children classified into the middle 60% of readers were classified as “Averagely Proficient”.

#### Passage Reading: AVI [Analyse van Individualiseringsvormen; AVI]

The AVI is an on-paper examination, aimed at monitoring the development of passage reading skills of children during Grades 1 to 6 of primary education (van Til et al., 2018b). AVI administrations were individually conducted and scored by teachers at the schools the children attended. During the AVI, children read Grade-level passages of increasing difficulty. The reading of passages continued until the child was unable to meet national norms, either by making too many mistakes, by not reading quickly enough, or by a combination of these factors. Then, based on the highest Grade-level passage read successfully, an AVI classification was provided. Specifically, the AVI categorizes children into one of twelve levels, ranging from the start to the end of primary education, providing an indication of the child’s progress throughout primary education. As these categories were provided by the school after specification, no reliability or validity information was collected. However, the reliability and validity of the AVI has previously been thoroughly investigated (Van Til., 2018b).

Based on the AVI classification, we specified three proficiency classes. Specifically, children who obtained an AVI categorization below the level of second grade were classified as “Less proficient”, while children with an AVI class at the level of the second or third grade were classified as “Averagely Proficient”. Children with an AVI classification above the third grade were classified as “Highly Proficient”.

### Argument-based Validation

The validation was implemented through the extended ABP (Kane, 1992, 2006, 2013; Wools et al., 2010). First, we specified the explicit inferences made about the decoding scores, by means of an IUA. Then, we described the validity arguments for each step of the IUA. Correspondingly, we specified the analyses conducted and evidence gathered for each validity argument. Finally, we evaluated the validity in its entirety, including the validation procedure.

Making the proposed interpretations and uses of test scores explicit was done through the specification of claims. For example, it could be claimed that the performance on a test provides a score that is observable. To evaluate this claim, warrants, rebuttals and backings were specified, which respectively concern statements that allow for the acceptance of the claim, evidence that refutes the claim or warrant, and evidence that supports the claim or warrant (Toulmin, 2003). It follows that the presentation of sufficient and qualitatively sound backings and warrants, alongside the justified rejection of rebuttals, leads to the acceptance of a claim.

**Fig. 1:**
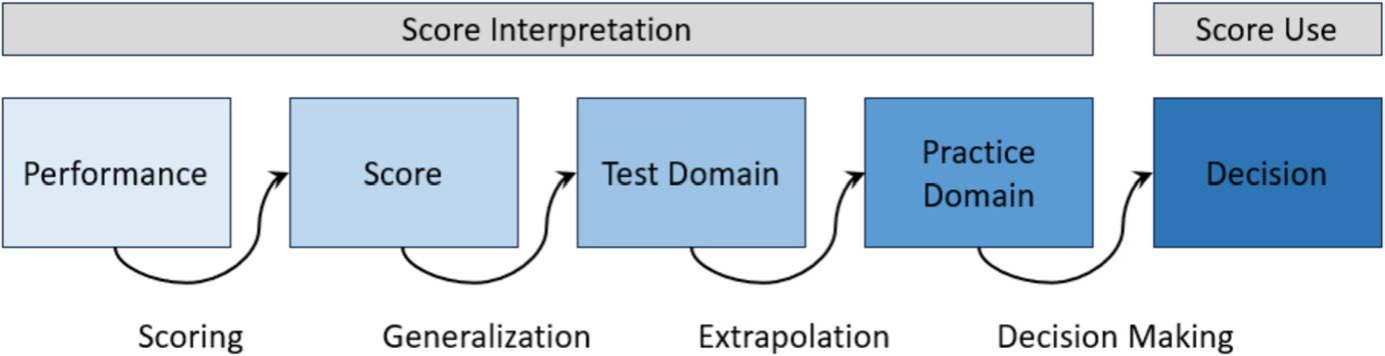
Interpretation and Use Argument (IUA) for the Validation

The IUA for the current study is presented in Fig. 1. Additionally, a detailed overview of the proposed inferences, assumptions, and sources of evidence is provided in Table 2. Correspondingly, the exact claims, warrants, rebuttals and backings for each inference are shown in Appendix A.

**Table 2:** Inferences, Assumptions, and Sources of Evidence Used to Validate the Reading Tasks

Inferences	Assumptions	Sources of evidence
Scoring: Performances on the reading tasks can be translated into observable and reliable ASR-based scores	- The scoring algorithm generates meaningful scores - The ASR-based word decoding and passage reading scores are reliable	- Evaluation of the ASR-based scoring algorithm through a comparison with human raters - Psychometric evaluation of the reliability of the word decoding and passage reading scores for all levels of ability and speed
Generalization: the oral reading scores, obtained over a limited sample of items and primary schools, can be used to make inferences about all possible samples of items and primary schools	- The tasks reflect relevant aspects of the learning goals and methods for oral reading in early primary education - The reading tasks reflect the difficulty level expected of oral reading tasks in Grades 2 and 3 - The sample of primary schools is representative of the population	- Compare the reading tasks to relevant learning goals and methods for early primary education in the Netherlands - Item difficulty parameters (mostly) cover the range of observed ability estimates - Compare the dialect region and school-weight distributions in the sample and population of primary schools
Extrapolation: The ASR-based oral reading scores can be used to make claims about children’s oral reading performance	- The reading tasks measure the same underlying construct as the DMT and AVI - The reading tasks are authentic, representing all relevant aspects of oral reading	- Correlate the LNIRT ability and speed estimates, and the person-level WCPM measures, with the DMT and AVI classifications - Evaluate whether the reading tasks provide information on oral reading accuracy, speed and automaticity
Decision Making: The ASR-based oral reading scores can be used to make decisions about proficiency and development	- The oral reading scores differentiate between good and less proficient oral readers - Misclassifications into highly, averagely and less proficient oral readers are minimized	- Evaluate the discrimination-parameters of all items - Predict DMT/AVI proficiency classes, using the oral reading scores

### Data Analysis

The analyses conducted throughout the present study were used to evaluate the validity arguments underlying the proposed inferences. All statistical analyses were conducted using RStudio (version 4.3.1; Posit Team, 2023).

#### Scoring Inference

To evaluate whether children’s performances on the reading tasks could be translated into observable and interpretable scores, we validated the ASR scoring-algorithm by comparing its item-level accuracy and speed scores to human annotations. In addition, we evaluated the reliability of the accuracy and speed scores.

To evaluate the validity of the item-level ASR-based accuracy and speed scores, we compared them with manual annotations. For the word decoding task, we only validated the accuracy scores, as the speed scores concerned logged data. Specifically, we obtained human annotations for 333 word decoding subtasks. These annotations were made by test leaders during task administration, using SERDA’s build-in test-leader app. Test leaders could label words as “read incorrectly”, while leaving them unlabelled marked them as read correctly.

For the passage reading task, test leaders reported that children read too fast to accurately annotate. Therefore, we obtained orthographic transcriptions of 18 subtasks, made by two Linguistics graduates. Transcribers respectively transcribed 12 and 9 subtasks, three of which were transcribed by both, in two tiers, using PRAAT (Boersma & Weenink, 2024). The first tier contains the prompts presented to the speaker. Each prompt is a word from the passage reading task, transcribed in an interval that contains all attempts a speaker made to read the prompt. The second tier contains the orthographic transcription of the audio, where each attempt to read a word was transcribed in a separate interval. If the last attempt in tier 2 was equal to the prompt word in tier 1, the prompt was labelled as read correctly. Meanwhile, the item-level speed scores were defined as the duration of the final reading attempt for a prompt.

Consistent with earlier studies on automatic accuracy assessment (Kheir et al., 2023), accuracy measures were encoded in terms of reading errors. Thus, correctly read words were labeled as False (i.e., word reading does not contain an error) and incorrectly read words as True (i.e., word reading does contain an error). Subsequently, we compared the ASR-based accuracy scores of both tasks to human annotations, using Matthew’s Correlation Coefficient (MCC). MCC yields a score between −1 and 1, where a score of 0 indicates that the correspondence is no better than chance. The MCC was chosen since our dataset is unbalanced, containing more correctly- than incorrectly read words, making the MCC a trustworthy and complete performance indication (Chicco et al., 2021). In addition, to enable a more thorough interpretation of these results, we computed the sensitivity (i.e., the proportion of decoding errors that are predicted to be incorrect), specificity (i.e. the proportion of actually correct readings that are predicted to be correct), and precision (i.e., the proportion of predicted incorrect readings that are actually incorrect).

Finally, to evaluate the passage reading speed scores, we computed correlations between human and ASR-based item-level speed scores. These are the correlations between the speed scores of transcriber 1 and the ASR, the speed scores of transcriber 2 and the ASR, and the speed scores of both raters and the ASR. However, the ASR is currently only able to produce speed scores for correctly read words. To elaborate, due to the large variation in possible reading errors that a child can make (e.g. repetitions at sub-word, word, phrase level or insertions of words that are not in the prompt), defining the desired output is very difficult. As a result, we only included speed scores for words that were read correctly.

To estimate the reliability of the ASR-based accuracy and speed scores we calculated Cronbach’s Alpha, Goodman’s Lambda 2 and the Greatest Lower Bound for the accuracy and speed measures of the word and passage reading tasks. In addition, we investigated the posterior standard deviations for children’s LNIRT ability and speed estimates. However, as the LNIRT model’s assumption of log-normal residuals was violated for most items, we replicated the generation of person- and item parameter estimates using a different IRT model, which only incorporates the item-level accuracy scores. The results, which can be found in Supplementary Appendix A, provided no indication that the violation of the log-normal residuals assumption substantially affected the results.

#### Generalization Inference

We assume that the sample of oral reading items can be used to make inferences about all samples of items if they reflect Dutch learning goals and methods for early primary education, if they match the difficulty level expected of oral reading tasks in Grades 2 and 3, and if the sample of primary schools represents the population.

To determine whether the reading tasks reflect Dutch learning goals and methods, the main argumentation of current guidelines was compared to the content of the reading tasks. The difficulty of the oral reading items was investigated by evaluating the distribution of the LNIRT item difficulty parameters and ability estimates. Finally, we evaluated the representativeness of the sample of primary schools by comparing the distribution of dialect region and school-weight to those found in the population.

#### Extrapolation Inference

The oral reading scores are assumed to provide information about oral reading performance if they measure the same underlying construct as their pen-and-paper predecessors, and if they provide information on oral reading accuracy, speed and automaticity.

To investigate whether the reading tasks measure the same construct as the DMT and AVI, we calculated correlations between the LNIRT ability and speed metrics, the person-level WCPM-scores, and the classifications of the DMT and AVI. The reading tasks’ authenticity was determined by discussing whether they reflect all relevant aspects of oral reading.

#### Decision Making Inference

It is assumed that children’s oral reading scores can be used to guide decisions regarding their performance if the reading tasks contain items that can discriminate between good and less proficient oral readers, and if misclassifications into highly, averagely and less proficient readers are minimized.

The discriminative ability of the reading tasks was determined by evaluating the LNIRT item discrimination parameters. To investigate the minimization of misclassification, we predicted the DMT and AVI proficiency classes through ordinal regression. Models included the LNIRT ability and speed estimates and the person-level WCPM scores of the word decoding and passage reading task. We also included children’s Grade, as the DMT and AVI are normed based on grade. Model performance was evaluated by calculating the weighted kappa with quadratic weights, and the average F1-score over all proficiency classes.

## Results

### Scoring Inference

To validate the ASR-based item level accuracy scores, we compared them to accuracy scores from human raters, using the MCC, sensitivity, specificity and precision. The results are presented in Table 3.

**Table 3:** Evaluation Metrics for the ASR-Based Item-level Accuracy Scores

Task	Subtasks	Items (inc, cor)	MCC	Sensitivity		Specificity	Precision
Word	333	16650 (2117, 14533)	0.43		0.93	0.69	0.31
Passage	18	3156 (542, 2614)	0.55		0.76	0.86	0.54

We found moderate agreement between human and automatic accuracy measures for the word decoding (MCC = 0.43) and passage (MCC = 0.55) reading task, and for the inter-rater agreement of the passage reading accuracy scores (MCC = 0.59). Additionally, the word (sensitivity = 0.93, specificity = 0.69) and passage (sensitivity = 0.76, specificity = 0.86) reading tasks showed moderate to high sensitivity and specificity. However, the word decoding task showed low precision (0.31), while the passage reading task showed moderate precision (0.54).

Then, we compared the ASR-based item-level speed scores of the passage reading task to their human equivalents. We found moderate to strong correlations between the ASR and transcriber 1 (*r* = 0.61), transcriber 2 (*r* = 0.57) and both transcribers (*r* = 0.59).

The reliability of the accuracy and speed scores was evaluated using Cronbach’s Alpha, Goodman’s Lambda 2 and the Greatest Lower Bound, and by evaluating the posterior standard deviation estimates from the LNIRT model for all LNIRT ability and speed estimates. Table 4 presents Cronbach’s Alpha, Goodman’s Lambda 2 and the Greatest Lower Bound for the word decoding and passage reading task. Reliability estimates ranged from 0.96 to 1, indicating that both tasks show excellent reliability.

**Table 4:** Cronbach’s Alpha, Goodman’s Lambda 2 and the Greatest Lower Bound for the Accuracy and Speed Measures of the Word Decoding and Passage Reading Tasks

Measure	Word Decoding		Passage Reading	
	Accuracy	Speed	Accuracy	Speed
Cronbach Alpha	0.96	0.99	0.99	0.99
Goodman’s Lambda 2	0.96	0.99	0.99	0.99
Greatest Lower Bound	0.98	1.0	1.0	1.0

**Fig. 2:**
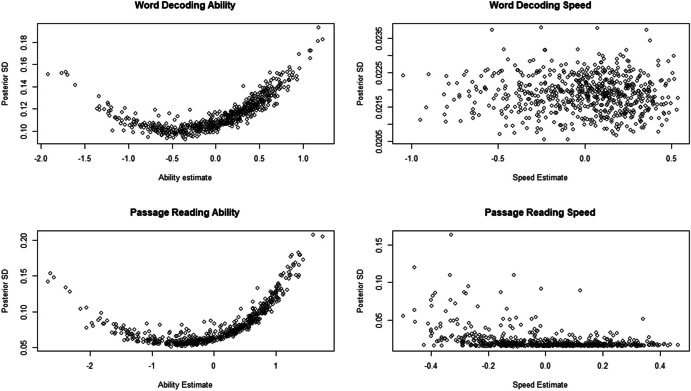
Posterior Standard Deviations for the LINIRT Word Decoding and Passage Reading Ability and Speed Estimates

Figure 2 shows the posterior standard deviation estimates for the LNIRT ability and speed estimates. Posterior standard deviations were relatively low, especially for the LNIRT speed estimates. Higher uncertainty was observed for relatively low and high ability estimates, and for a handful of negative passage speed estimates.

### Generalization Inference

To evaluate the generalizability of the oral reading scores, we compared the content of the reading tasks to current Dutch learning goals and methods for early primary education, and compared the distributions of the LNIRT ability- and item-difficulty estimates. In addition, we compared the sample of primary schools to their population with regard to dialect region and school weight.

Dutch can be considered a semi-transparent language because of its relatively consistent grapheme-phoneme correspondences (Borgwaldt et al., 2004; Seymour et al., 2003). Correspondingly, learning to read is primarily based on grapheme-phoneme rules. Accordingly, most schools in the Netherlands use a reading instructional method (mostly either Veilig Leren Lezen [learning to read safely; (Zwijsen Educatieve Uitgeverij, 2023)] or Lijn 3 [Track 3; (Malmberg, n.d.)] that focusses on these rules to instruct decoding. These methods focus on teaching children grapheme-phoneme correspondences (including digraphs such as *ei, ui* and *ou*) and on learning to read simple structured words (both mono- and bi-syllabic) throughout the first half of Grade 1. Then, focus gradually shifts towards automatizing the reading process and reading more complex structured words (e.g. consonant clusters and bi-/polysyllabic words). After Grade 1, schools either use a method for advanced decoding instruction, incorporate it in instruction for other language-related instruction (e.g., reading comprehension of language arts), focus on furthering the automatization of the reading process, and/or focus instruction on advanced reading difficulties (e.g., loanwords and the use of *c, x* and *y* in words).

Likewise, SERDA’s reading tasks build up in difficulty over its subtasks. Specifically, the first word decoding subtask primarily contains one-syllable words with various consonant–vowel combinations and relatively basic reading difficulties (e.g. open syllable, sch-). Meanwhile, the second subtask focusses on one-, two- and three syllable words with more advanced reading difficulties (e.g. ge-, -lijk), while the third subtask contains two-to-four syllable words with more complex reading difficulties (e.g. -isch, loanwords). The same can be said for the passage reading task, which focusses on reading mono-, bi- and polysyllabic words with an increase in orthographic inconsistencies and complexities over subtasks. Finally, conform the instructional methods discussed, the reading tasks place specific focus on automatizing the reading process through a focus on both accuracy and speed in instruction and performance metrics alike. Altogether, the reading tasks closely match the way in which children are taught how to read in the Netherlands.

**Fig. 3:**
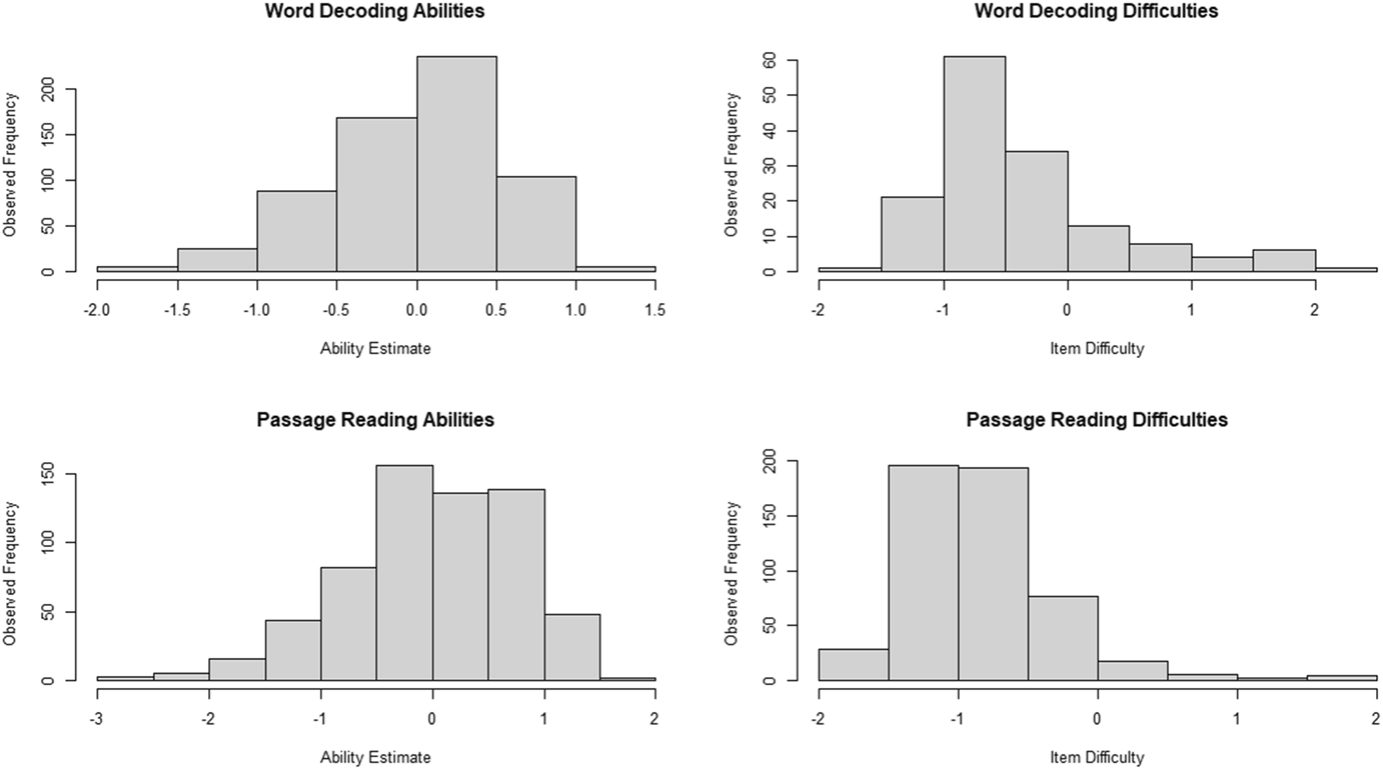
LNIRT Ability and Difficulty Estimates for the Word Decoding and Passage Reading Task

Figure 3 shows the distribution of the LNIRT ability- and item-difficulty estimates of the word decoding and passage reading task. For both tasks, the item difficulty parameters cover the range of ability estimates, with the exception of some extremely low passage reading ability estimates. However, the difficulty of the items is generally low, showing relatively few items with positive difficulty estimates.

**Fig. 4:**
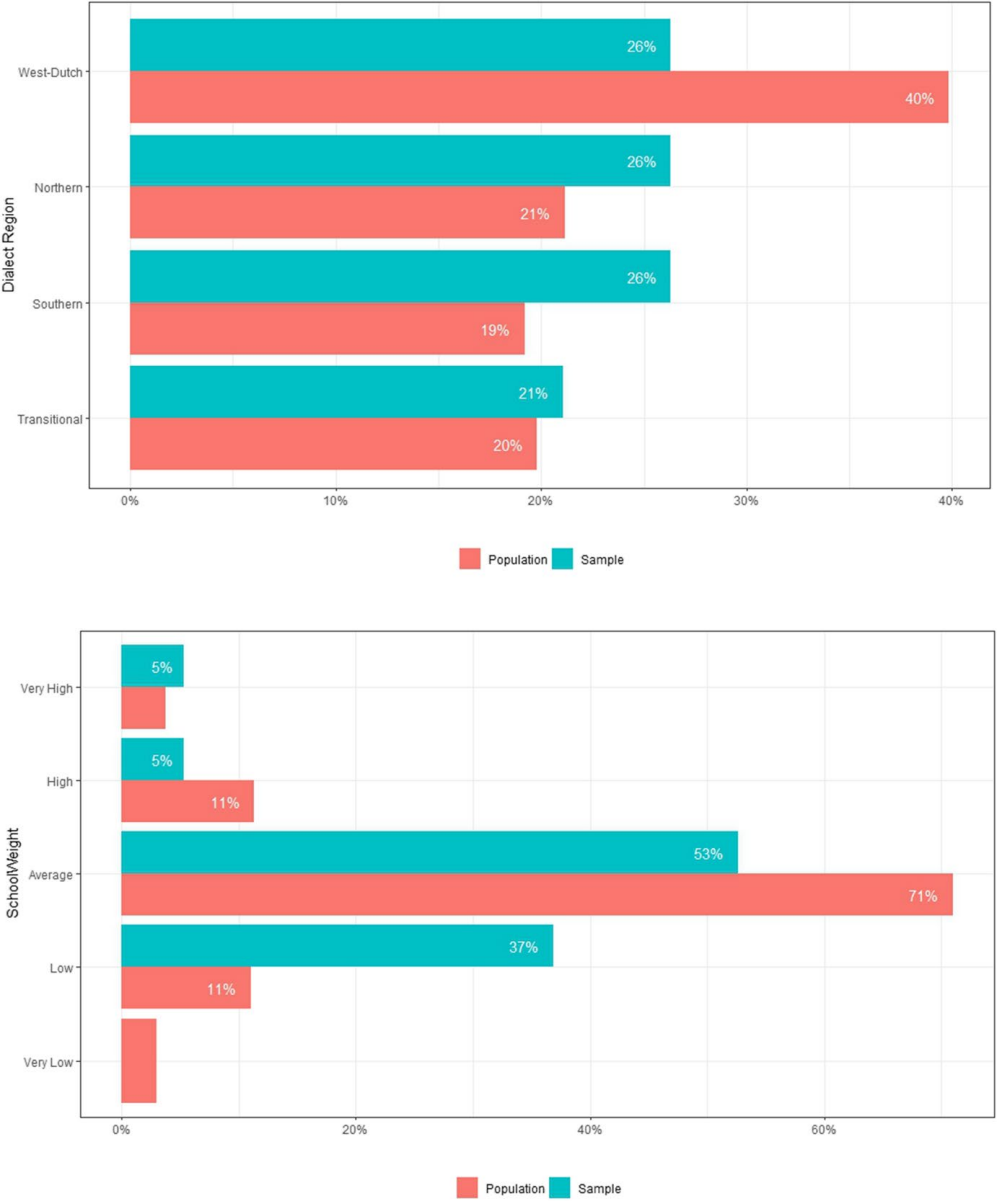
Sample and Population Distributions for Dialect Region and School Weight

Figure 4 shows the dialect region and school-weight distributions for the population and sample of primary schools in the Netherlands. Generally, the sample resembles the population. However, an underrepresentation of schools in the Western dialect region was observed, while the sample overrepresents schools with low school-weights.

### Extrapolation Inference

To evaluate whether the oral reading scores allow for claims about oral reading performance, we calculated correlations between the LNIRT ability and speed estimates, the person level WCPM scores, and the categories of the DMT and AVI. In addition, we evaluated whether the word decoding and passage reading tasks provide information on all aspects of oral reading.

Table 5 presents the correlations between the ability estimates of the word decoding and passage reading task, the person level WCPM measures, and the categories of the DMT and AVI. Correlations varied between 0.32 and 0.88, showing weak to very strong correspondences between the oral reading metrics.

**Table 5:** Pearson and Spearman Correlations Between the LNIRT Ability and Speed Estimates, the Person Level WCPM Metrics, and the Categories of the DMT and AVI

Metric	Ability Word	Speed Word	Ability Passage	Speed Passage	WCPM Word	WCPM Passage		DMT	AVI
Ability Words	1	-	-	-	-	-	-		-
Speed Word	0.65	1	-	-	-	-	-		-
Ability Passage	0.64	0.50	1	-	-	-	-		-
Speed Passage	0.61	0.75	0.43	1	-	-	-		-
WCPM Word	0.87	0.88	0.56	0.74	1	-	-		-
WCPM Passage	0.68	0.77	0.70	0.86	0.79	1	-		-
DMT	0.40	0.57	0.32	0.60	0.54	0.67	1		-
AVI	0.55	0.69	0.42	0.78	0.68	0.80	0.65		1

Correlations with the DMT and AVI were Spearman correlations, while the others concerned Pearson correlations. All correlations were significant at a = 0.001

To reflect oral reading skills, the reading tasks should provide information regarding children’s oral reading accuracy, speed and automaticity. Table 1 shows that the word and passage reading tasks provide information on oral reading accuracy, speed and automaticity (WCPM). Meanwhile, the validation has shown that item-level information is reliable and that most of the resulting child-specific metrics moderately to strongly resemble current oral reading metrics. Therefore, we argue that child-specific information is provided on children’s oral reading accuracy, speed and automaticity through, respectively, the LNIRT ability and speed estimates, and the WCPM metrics.

### Decision Making Inference

To evaluate whether the oral reading items differentiate between good and less proficient oral readers, we evaluated the LNIRT item discrimination parameters. In addition, we investigated the classification accuracy of ordinal regression models that predicted oral reading proficiency, using the LNIRT ability and speed estimates, the person level WCPM metrics, and children’s Grade.

**Fig. 5:**
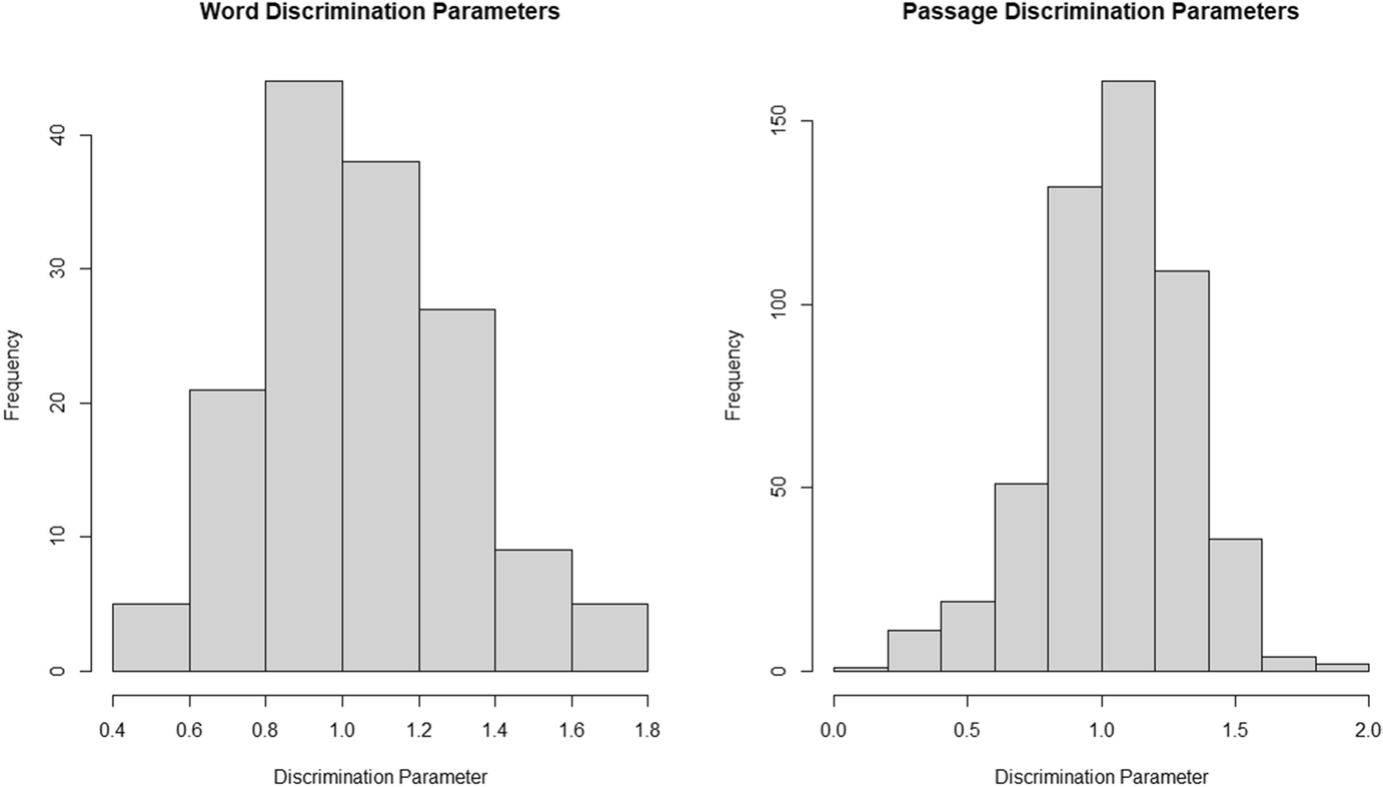
LNIRT Item Discrimination Parameters for the Word Decoding and Passage Reading Task

Figure 5 shows the item discrimination parameters of the LNIRT model for the word decoding and passage reading task. Discrimination parameters primarily ranged between 0.8 and 1.4 for both the word and passage reading task, indicating that items generally discriminate moderately well or better (Baker, 2001; Bichi & Talib, 2018). Lower discrimination parameters were primarily observed for single-syllable words and articles.

The ordinal regression model used to predict the DMT proficiency classes showed a weighted kappa of 0.65 and an average F1 score of 0.70, indicating moderate to good classification accuracy. The ordinal regression model used to predict the AVI proficiency classes showed a weighted kappa of 0.73 and an average F1 score of 0.77, indicating moderate to good classification accuracy.

### Validation Evaluation

Following the presentation of validity evidence, we applied the ABP (Kane, 1992, 2006, 2013) to evaluate whether the proposed interpretations of SERDA’s reading tasks can be substantiated. For these purposes, we used the three criteria specified by Wools et al. (2010) to validate the validation process in its entirety.

The first criterion focusses on the complexity of the interpretive argument, as evidenced through the number of inferences and their level of detail. To meet the first criterion, at least four inferences should be specified, each including at least one backing, warrant and rebuttal. As shown in Appendix A, the current study specified four inferences, which were all supplied with at least one backing, warrant and rebuttal. Therefore, the interpretive argument is deemed sufficiently complex and detailed, allowing for the acceptance of the first criterion.

The second criterion focusses on evaluating the presented evidence with regard to plausibility and coherence. For each inference, the validity argument is evaluated in full, resulting in the acceptance or rejection of the inference.

For the scoring inference, we found that the ASR-based oral reading scores resemble human ratings moderately well. In addition, the reliability of the word and passage reading tasks was deemed excellent, showing high internal consistency and low posterior standard deviations for most ability levels. Therefore, the validity evidence substantiates the scoring inference sufficiently to warrant its acceptance.

For the generalization inference, the gathered validity evidence indicates that the reading tasks reflect the way in which children learn to read in the Netherlands, while the difficulty of the tasks matches the expected difficulty in Grade 2 and Grade 3 of primary education. In addition, although some differences with the population were observed, the sample of schools generally represented the population well with regard to the distribution of dialect region and school-weight. Therefore, the validity evidence sufficiently substantiates the acceptance of the generalization inference.

For the extrapolation inference, we found that most LNIRT ability estimates, all LNIRT speed estimates, and the person level WCPM metrics, resembled the DMT and AVI classifications moderately well or better. In addition, we argued that the reading tasks provide item and person level information that reflect oral reading accuracy, speed and automaticity. Based on these results, we conclude that the reading tasks provide scores that reflect oral reading skills, thereby justifying the acceptance of the extrapolation inference.

For the decision making inference, the discrimination parameters from the LNIRT model suggest that most items are able to discriminate between good and less proficient oral readers moderately well or better. In addition, we found moderate to good classification accuracy when predicting the DMT and AVI proficiency classes with the decoding metrics. Therefore, the evidence indicates that the decision making inference is validly assumed, leading to its acceptance.

The third criterion emphasizes the plausibility of the validity argument as a whole, thereby taking into account all validity evidence of all inferences. Accordingly, the third criterion can only be justified if the first two criteria have been met. Based on the acceptance of the first criterion, and each step of the second criterion, the third criterion is also deemed justified. Thus, given that sufficiently numerous and detailed validity evidence has been presented to deem each of the specified inferences plausible, the validation as a whole is also deemed plausible.

## Discussion

The aim of the current study was to investigate whether valid word decoding and passage reading metrics could be generated for a semi-transparent language, using an ASR-based oral reading fluency assessment instrument. The validation was conducted using the extended ABP (Kane, 1992, 2006, 2013; Wools et al., 2010), which includes a validation evaluation. Subsequently, we present the interpretation of the results, suggestions for future research, and the conclusion.

Based on the results of the validation, the scoring inference was deemed plausible, indicating that oral reading performances can be reliably translated into observable ASR-based oral reading scores. However, while the reliability of the ASR-based scores was excellent, the ASR-based scores showed only moderate resemblance to human raters. To be more specific, both tasks showed high sensitivity and specificity, but only moderate to low precision was observed, indicating that the ASR somewhat overestimates the number of errors a reader makes.

The lower precision could be explained through the unbalanced state of the data, as most items were read correctly. Given that reading errors were the clear minority group, and given that errors were coded as positives, false positive classifications occurred most frequently, leading to lower precision. As this unbalance was more prominently the case for the word decoding task, its precision was especially affected. More generally, a possible explanation for these moderate results is that state-of-the-art ASR models (as used in this study) are trained on adult speech, and therefore perform worse on child speech, which shows more variability than adult speech (Feng et al., 2024). Child speech recognition is a challenging problem, mainly because of the lack of annotated data to capture speech variability. In future research, we would like to extent the SERDA speech corpus with annotations, so that they can be used to improve child speech recognition. Based on the moderate agreement and low to moderate precision, even though the current study has shown evidence that observable and reliable scores can be generated using an ASR-based approach, we suggest against immediate usage in high-stakes settings.

While these limitations also argue against the acceptance of the scoring inference, the purpose of this validation was not to evaluate whether ASR-based oral reading metrics can be used in high stakes settings, nor whether each individual ASR prediction is correct. Instead, we evaluated whether observable ASR-based oral reading scores could be generated such that practitioners can be reliably informed about children’s oral reading skills. In short, while future researcher should focus on optimizing ASR performance for this speech corpus, and ASR based on children’s speech in general, the observed results were deemed sufficient to satisfy the scoring inference within more formative test settings, matching SERDA’s developmental purposes (van der Velde et al., 2024b).

The investigation of the generalization inference resulted in its acceptance, suggesting that the sample of oral reading items and primary schools represent the population. However, a potential issue concerned task difficulty, as many items were shown to be relatively easy. An explanation could be found in the relatively large number of primary schools with a low school-weight. To elaborate, a low school-weight indicates that the children attending a school, on average, are expected to perform relatively well compared to the population. In other words, the prevalence of well-performing schools could have resulted in a sample with relatively many well-performing children, for whom the items are relatively easy. Thus, although the results provide evidence that generalisations towards the general population of second and third graders is possible, further investigations are required into both the performance of the ASR, and the resulting scores, in samples of predominantly highly and less skilled oral readers.

The evaluation of the validity evidence resulted in the acceptance of the extrapolation inference, indicating that the reading tasks reflect oral reading skills. Specifically, we found that most LNIRT ability and speed estimates were strongly related to the person level WCPM metrics. With regard to the DMT and AVI, the speed estimates and WCPM metrics outperformed the ability estimates. This finding is unsurprising, given that earlier research has stressed the importance of variability in reading speed, and therefore automaticity, throughout reading development (e.g. Verhoeven & van Leeuwe, 2009), while accuracy tends to show lower variability between children as they progress through primary education. To conclude, the results support the assumption that the reading tasks measure oral reading skills, while simultaneously highlighting the importance of including the assessment of speed when using ASR to measure oral reading fluency.

The decision making inference was also justified, showing that the reading tasks allow users to make decisions regarding children’s oral reading proficiency and development. However, some of the passage reading items did not have much, or any, discriminative ability. Investigations unearthed that these items primarily concerned articles (e.g. “a”, “an” [*een, de, het]*), and other single-syllable words, which contain little to no orthographic complexity. Although an investigation into LNIRT model performance without these items could be of interest, this would also reduce the amount of items in the passage reading task, potentially making the passage reading task more difficult. Therefore, this consideration should be more thoroughly investigated before it is implemented.

Based on these findings, the following recommendations for further research are specified. Firstly, while the present study has demonstrated promise regarding the use of ASR to assess oral reading fluency skills for young children, researchers are advised to investigate the optimalization of ASR performance for the current speech corpus, including an evaluation of the Word Error Rate (WER). Especially interesting would be an assessment of ASR performance for samples containing primarily highly or less proficient orally fluent readers. When compared to the results of the current study, such investigations can provide a more thorough understanding of the behaviour of the ASR, and the resulting LNIRT item parameters and person estimates, potentially leading to higher ASR performances on children’s speech in general. Secondly, the performance of the LNIRT model should be compared for different subsets of items, allowing for the specification of an optimal, or minimally required, set of items or subtasks. Researchers are advised to focus on the evaluation of models that exclude items with low to no discrimination ability, and on a more specific evaluation of the word and passage decoding subtasks. Finally, now that it has been shown that oral reading metrics can be extracted from SERDA, work should focus on including and validating a prosody component.

## Conclusion

In conclusion, the gathered evidence suggests that valid word decoding and passage reading measures can be generated for a semi-transparent language, using an ASR-based oral reading fluency assessment instrument. The results provide evidence that the reading tasks can be used to obtain observable and reliable oral reading metrics, while the samples of reading tasks and Dutch primary schools were deemed plentiful and representative enough to warrant generalizations towards their general populations. Evidence also substantiated that the reading tasks measure oral reading skills, and that the tasks allow users to make some claims and decisions regarding children’s oral reading proficiency and development. However, the ASR requires optimalization and its errors require further exploration through the analysis of, for example, Word Error Rates. Furthermore, generalizations towards high and low proficiency populations should be more thoroughly investigated, allowing for comparisons of ASR performance, and LNIRT item and person characteristics behavior, such that ASR performance for children’s speech can be improved. Finally, future researchers are advised to realize and validate a prosody component. If implemented correctly, these changes would complete the envisioned oral reading fluency assessment instrument, thereby improving the provision of detailed diagnostics, reducing teacher’s testing burden, and improving the assessment of oral reading fluency throughout all of primary education.

## Supplementary Information

Supplementary Material 1.

Supplementary Material 2.

## Data Availability

The data used throughout the study are available from the corresponding author upon reasonable request.
