# Supplementary material for: Speech Enabled Reading Fluency Assessment: a Validation Study
Source: Int J Artif Intell Educ. 2025 May 14;35(4):2569–95. doi: 10.1007/s40593-025-00480-y (PMC12686063; doi:10.1007/s40593-025-00480-y)

# **Appendixes**

## **Appendix A: Claims, Warrants, Rebuttals and Backings for Each Inference**

Followingly, the claims, warrants, rebuttals and backings for each inference are visually presented. At the top of each appendix, a description is provided for the current inference (followingly referred to as claim). The claim, corresponding to the current step in the IUA, is then shown in block notation (e.g. from performance to score in Appendix A1). Uninterrupted lines moving towards a block or line indicate a warrant, which substantiates the claim if it is found to be true, or a backing, which substantiates a warrant if it is found to be true. Interrupted lines moving towards a line concern a rebuttal, which substantiate the rejection of the warrant or backing it is linked to, if it is found to be true.

The argumentation presented within the appendixes is hierarchical, working up from the bottom. In practice, this means that the statements from the blocks at the bottom of the graphs precede and inform statements made higher up. In ABP terminology, these lower blocks contain the backings and rebuttals for warrants, while the graphically higher blocks concern warrants and rebuttals for claims. For example, in Appendix A1 the evaluation of the reliability of the word decoding and passage reading performances is preceded by the evaluation of the ASR-scoring algorithm. This dependency follows from the reasoning that, if the ASR-scoring algorithm provides nonsense, then it does not matter whether this nonsense is reliably provided. In that case, whatever the result of the reliability analysis might be, the warrant can not be accepted. In other words, if the ASR-scoring algorithm does not perform satisfactorily, the backing for the warrant “word decoding and passage reading performance can be reliably estimated” is rebutted, leading to the rejection of the claim.

### ***Appendix A1: Scoring Inference***


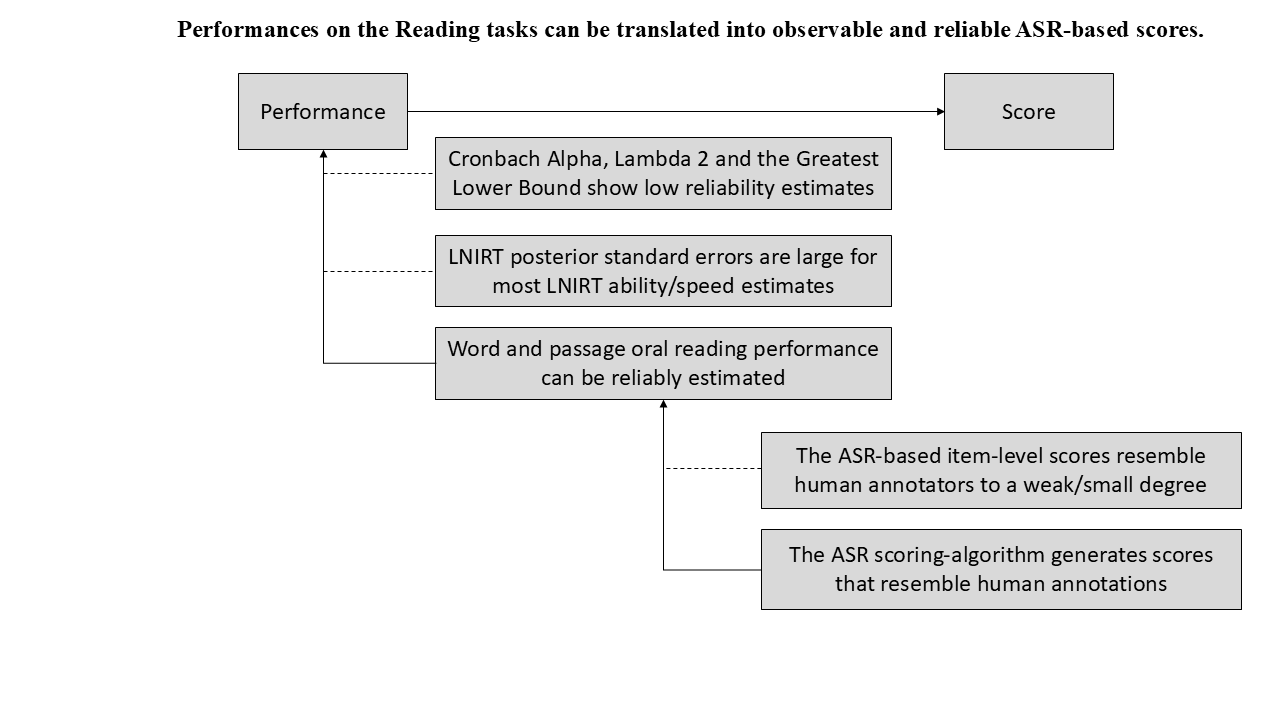


### ***Appendix A2: Generalization Inference***


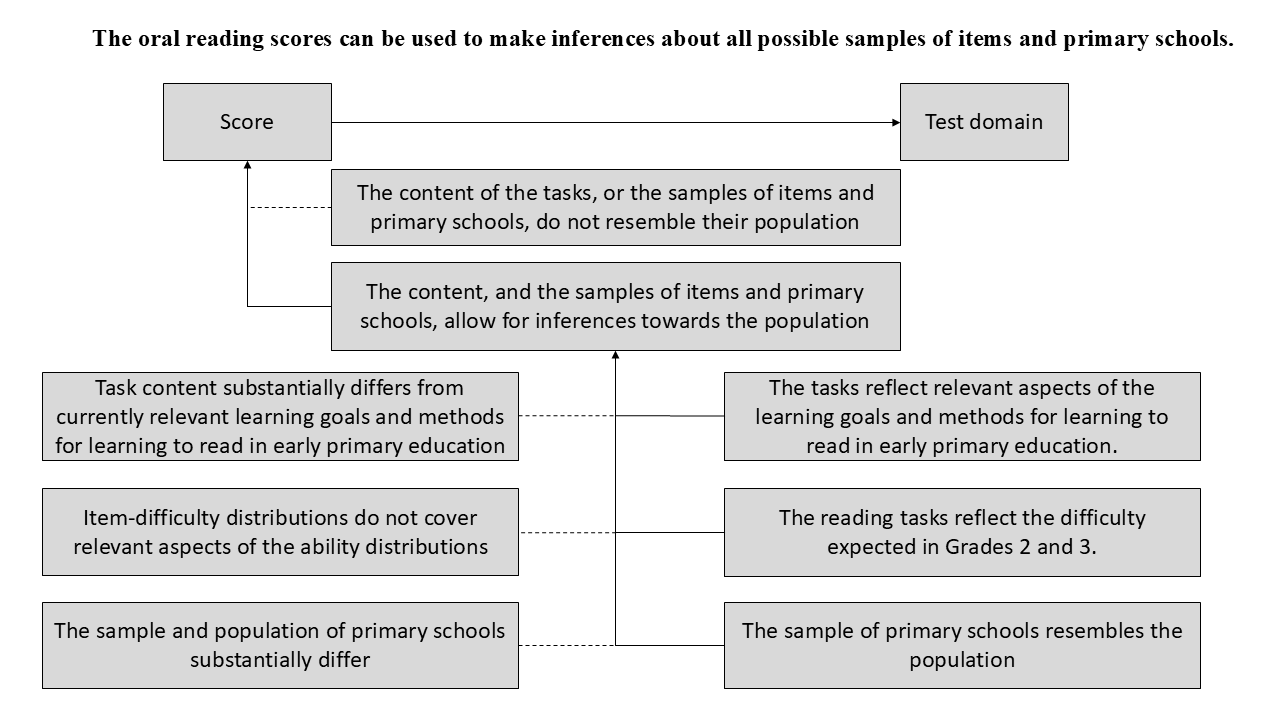


### ***Appendix A3: Extrapolation Inference***


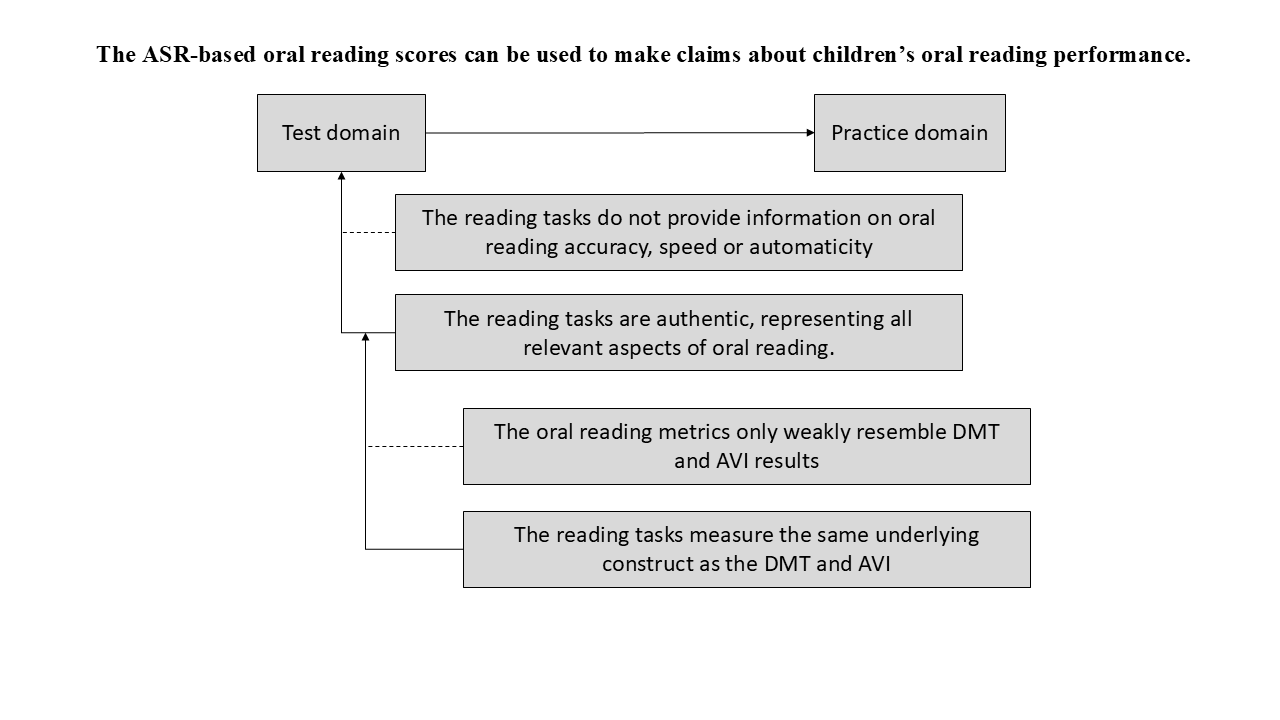


### ***Appendix A4: Decision Making Inference***


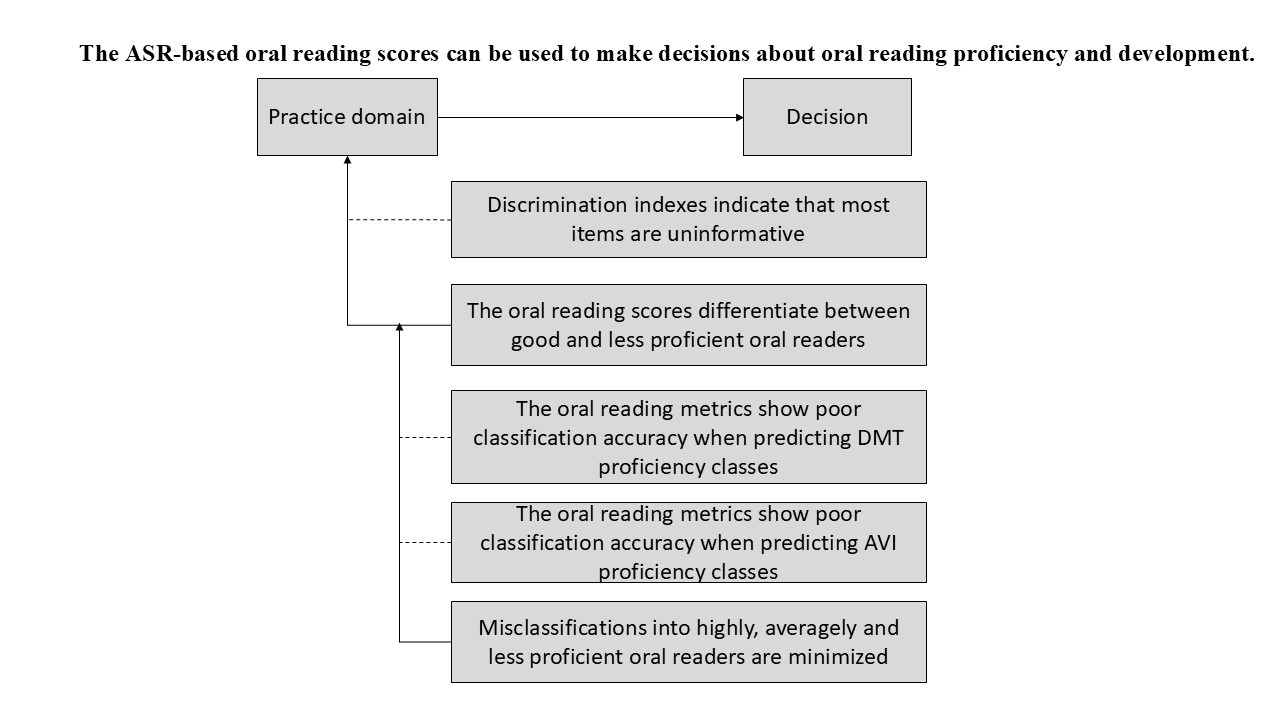

Supplement: Supplementary file 1 — Supplementary Material 1. [file 40593_2025_480_MOESM1_ESM.docx]
