# Supplementary material for: Speech Enabled Reading Fluency Assessment: a Validation Study
Source: Int J Artif Intell Educ. 2025 May 14;35(4):2569–95. doi: 10.1007/s40593-025-00480-y (PMC12686063; doi:10.1007/s40593-025-00480-y)
Supplement: Supplementary file 2 — Supplementary Material 2. [file 40593_2025_480_MOESM2_ESM.docx]

# **Supplementary Appendix A: LNIRT vs 2pl**

# Article information

Title: Speech Enabled Reading Fluency Assessment: a Validation Study

Journal: International Journal of Artificial Intelligence in Education

Authors:

Max van der Velde^1,2^ , Wieke Harmsen^4^, Bernard P. Veldkamp^1^, Remco Feskens^1,2^, Jos Keuning^2^, Nicole Swart^3^

^1^Cognition, Data and Education section, Faculty of BMS, University of Twente, Enschede, The Netherlands

^2^CitoLab, Cito, Arnhem, The Netherlands

^3^Expertisecentrum Nederlands, Nijmegen, The Netherlands

^4^Centre for Language Studies, Radboud University, Nijmegen, The Netherlands

Corresponding author: Max van der Velde, [m.e.vandervelde@utwente](mailto:m.e.vandervelde@utwente)

# Appendix content

Throughout this appendix, we compare the distributions of the person and item parameters extracted from the LNIRT model to those extracted from a 2-parameter logistic (2PL) IRT model, which was specified using the MIRT R-package. Specifically, we compare the posterior standard deviation of the LNIRT model to the standard errors of the 2pl model. Then, the distributions of the person ability estimates, and the item- difficulty and discrimination parameters, are compared.

## **Comparison 1: 2PL Standard Errors vs LNIRT Posterior Standard Deviations**

### ***Words***


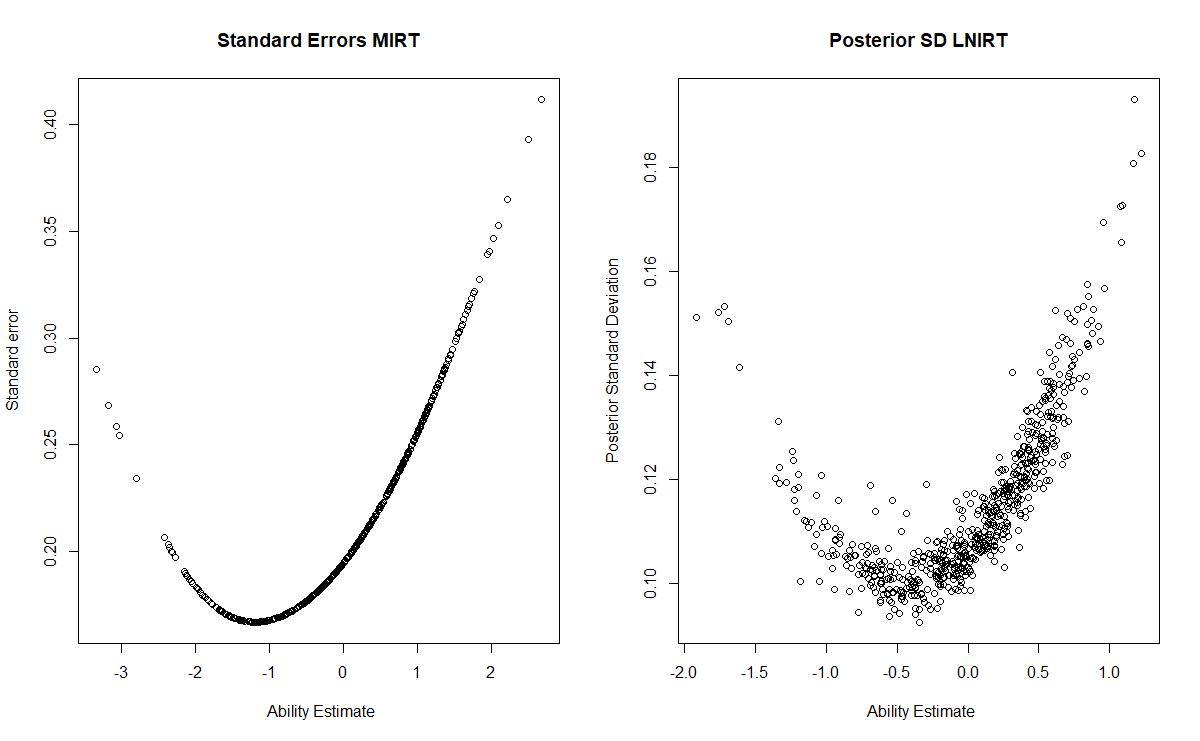


### ***Stories***


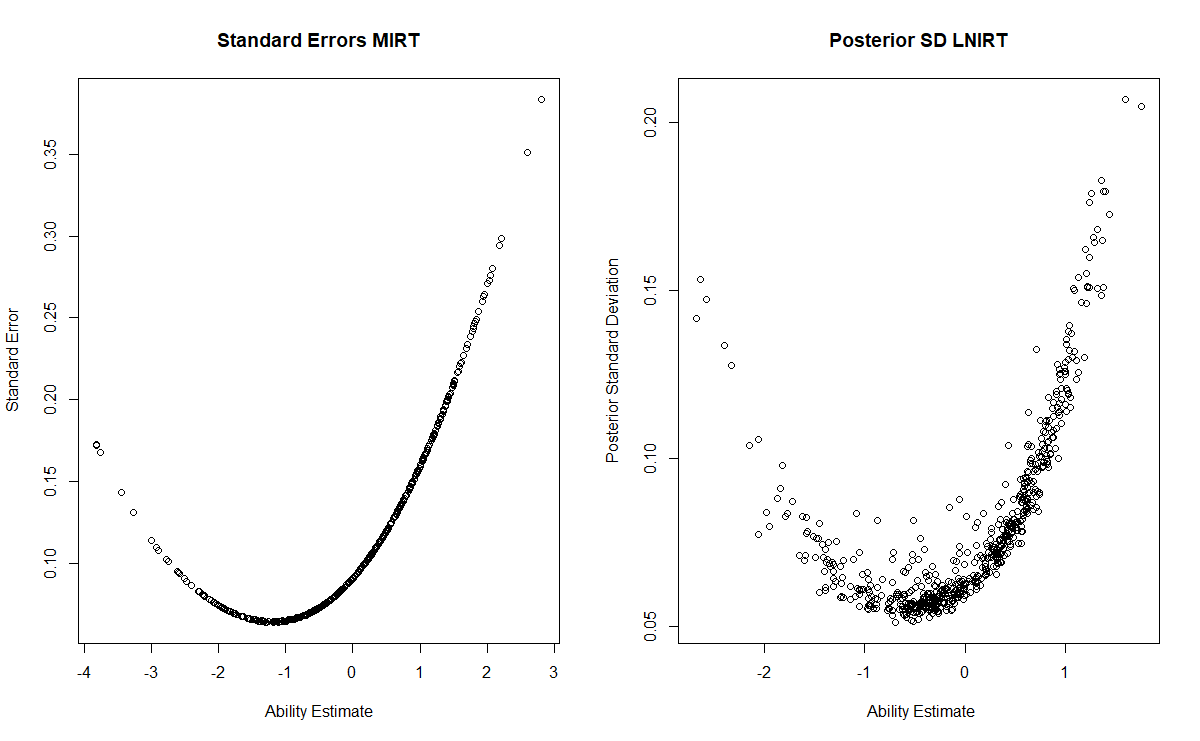


Both graphs show similar error distributions, although the standard errors for the MIRT model are higher, in general, than the LNIRT posterior standard errors. It should be noted, however, that the scale of the ability estimates differs somewhat for both models, which corrects for some of these differences. In addition, we see that the LNIRT model has higher variability between persons with the same ability estimate, while the MIRT model has the same error for persons with the same ability. In general, the distributions do not differ in a relevant manner between models, providing little evidence that the models perform differently.

## **Comparison 2: Ability Distributions**

### ***Words***


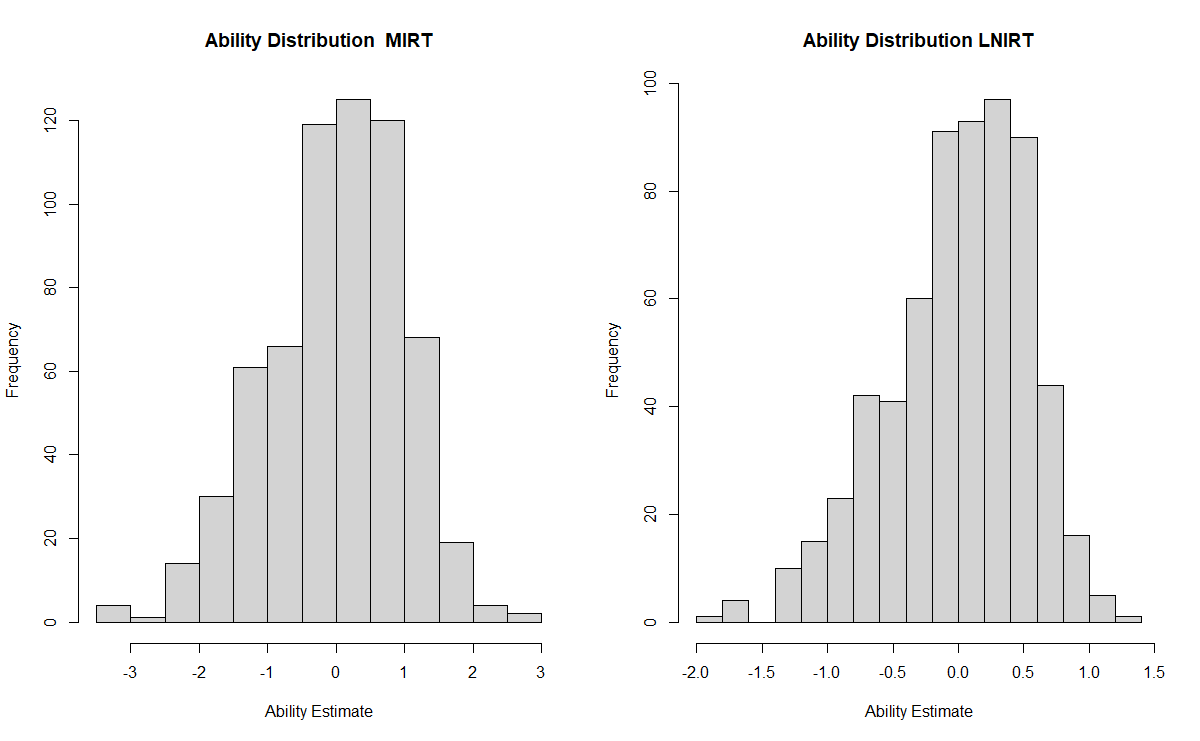


### ***Stories***


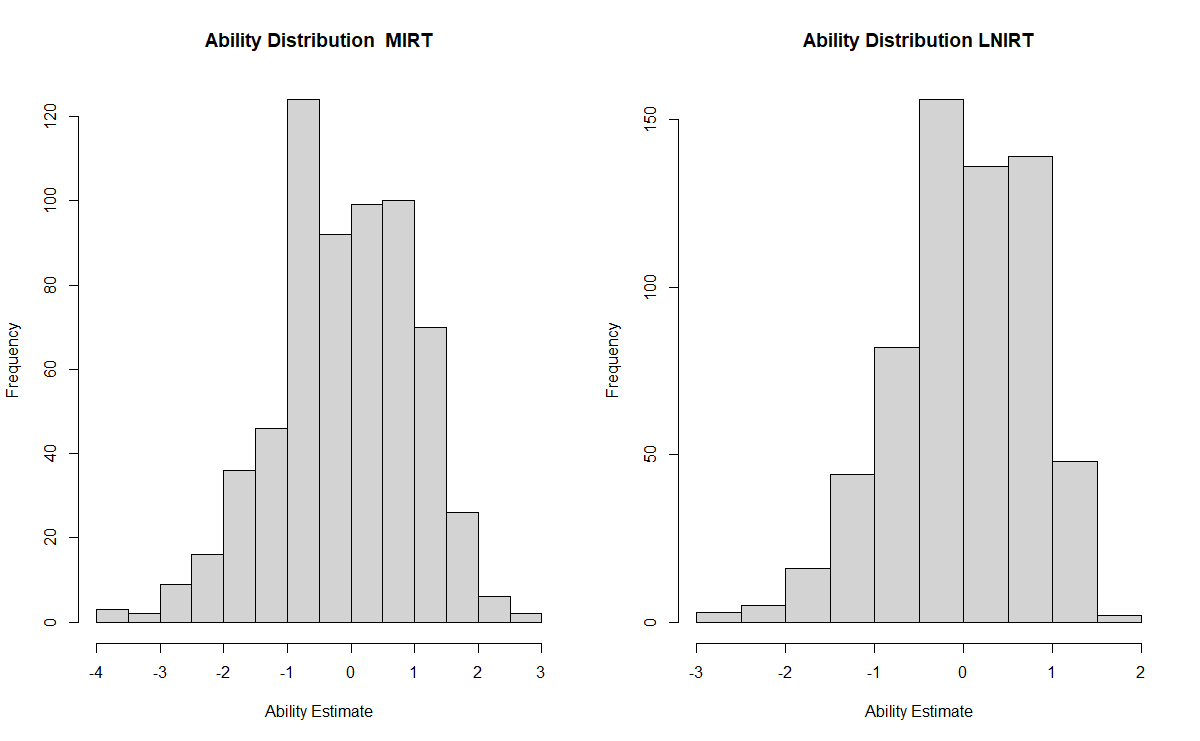


The distribution of the ability estimates of the MIRT and LNIRT IRT models resemble one another almost perfectly, showing correlations of 0.998 and 0.997 for, respectively, the word and passage reading task. The distributions look quite similar, with the only meaningful difference between the models, once more, concerning the scale of the ability estimates.

## **Comparison 3: Item Difficulty and Discrimination**

### ***Words***


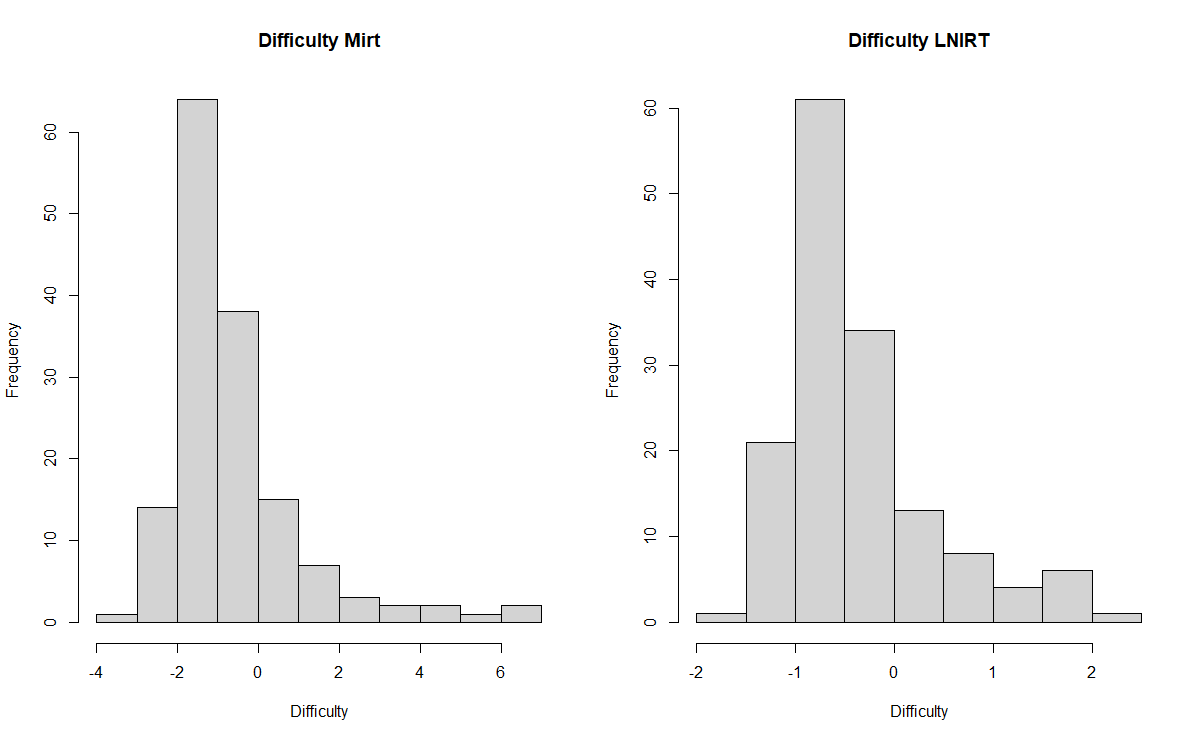


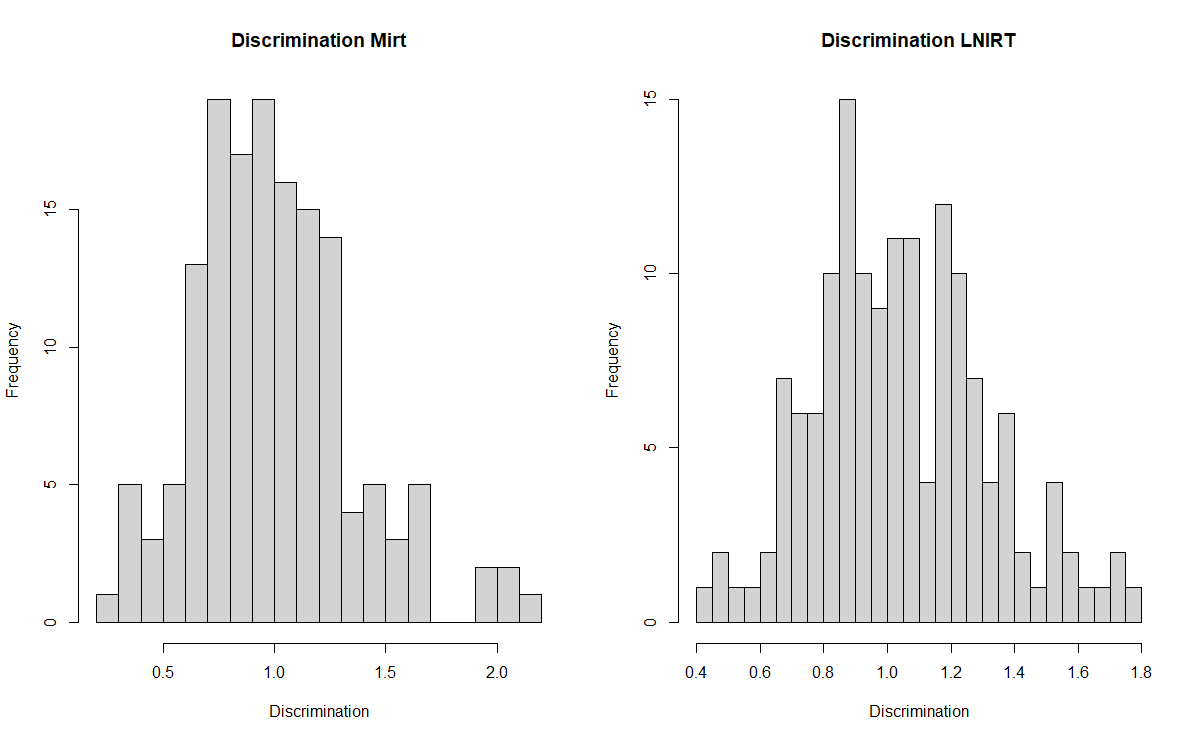


### ***Stories***


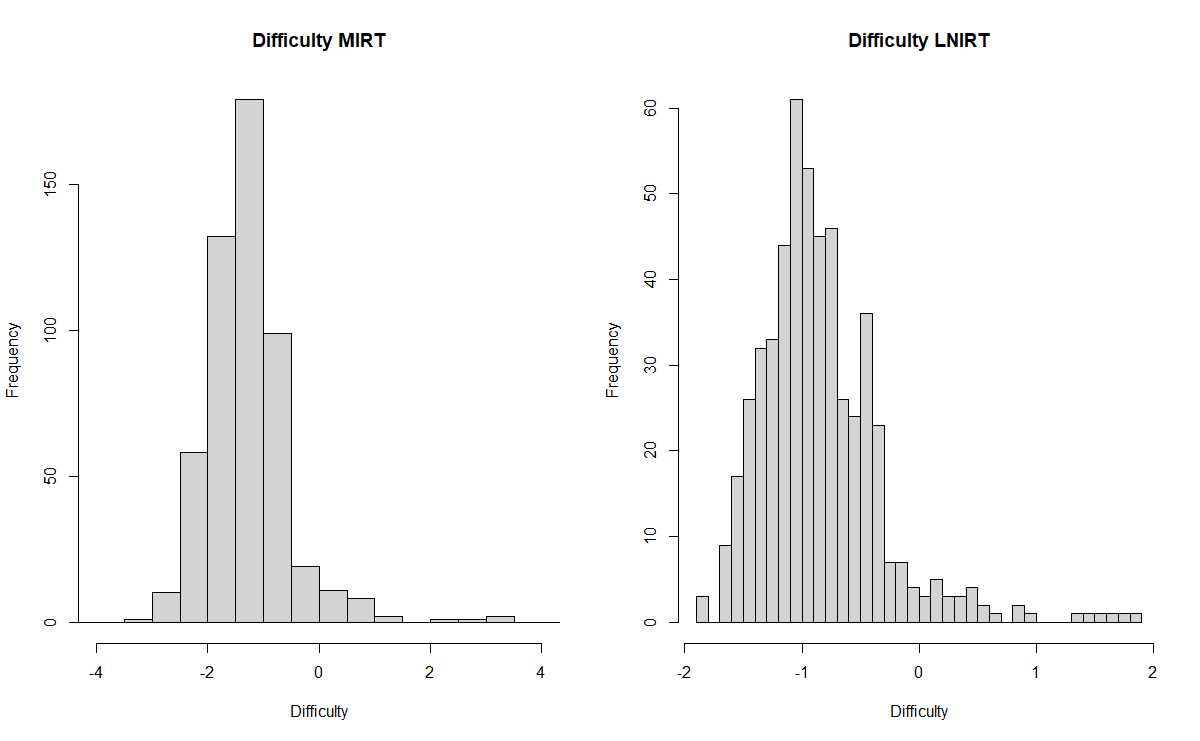


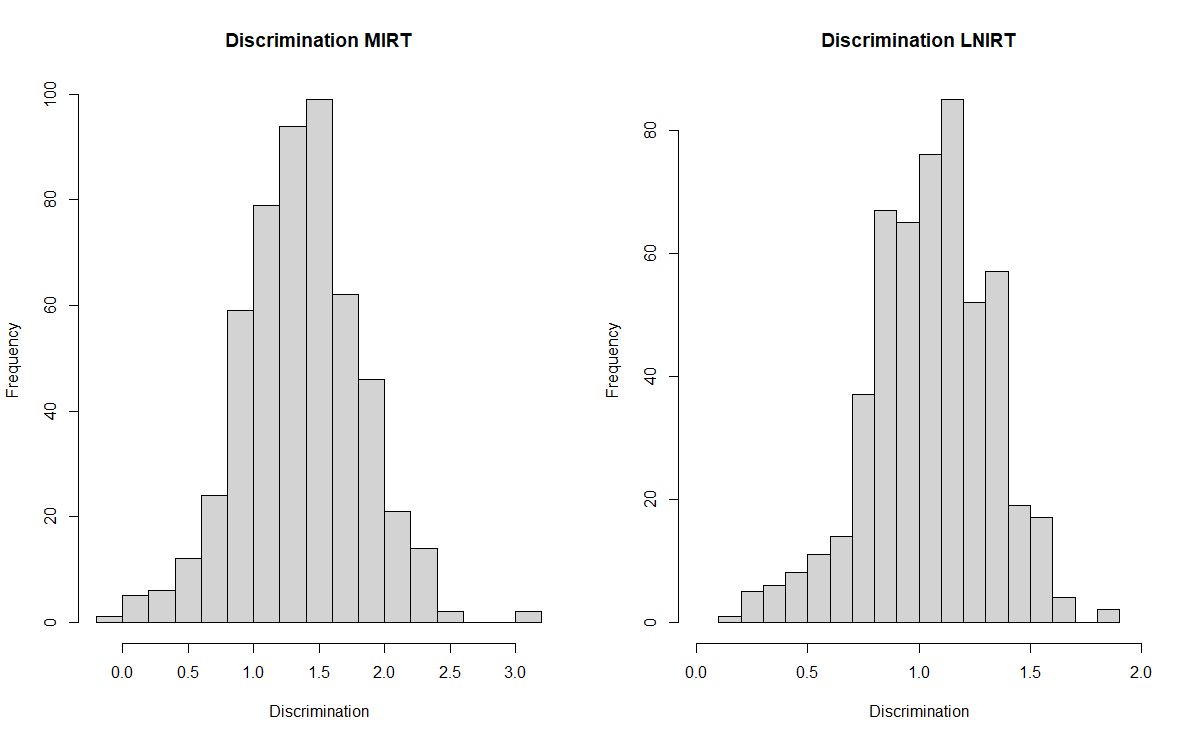


Like the error and ability estimate comparisons, the distributions of the item difficulty and discrimination parameters resemble one another to a high degree. For the word decoding tasks, the correlation between the difficulty parameters was 0.98, while the correlation between the discrimination parameters came down to 0.97. For the passage reading task, the correlation between the ability estimates was quite a bit lower (0.49), which can mostly be attributed to the existence of some extremely large (10+) and small (-20 and smaller) ability estimates (left out of the graph, for the sake of comparison clarity), which is a relatively well known issue with 2PL models that follow the standard IRT parameterization. The correlation between the discrimination parameters was 0.99 for the passage reading task, showing high resemblance. With regard to the distributions of the discrimination parameters, the MIRT model shows higher values, indicating somewhat higher informativeness. However, no substantial differences can be inferred from these comparison for either the difficulty, nor the discrimination parameters.

## **Conclusion**

Given the similarity between the distributions of the errors, the person and item parameters, and the observed correlations between the model parameters, we deem it unlikely that the violation of the log-normality assumption severely impacts the results for accuracy-based estimates of the word decoding or passage reading task. Therefore, we deem it acceptable to apply the LNIRT IRT model throughout the current study.
